# Body image mediates the depressive effects of weight gain in new mothers, particularly for women already obese: evidence from the Norwegian Mother and Child Cohort Study

**DOI:** 10.1186/s12889-016-3363-8

**Published:** 2016-07-29

**Authors:** Seung-Yong Han, Alexandra A. Brewis, Amber Wutich

**Affiliations:** 1 Mayo Clinic/Arizona State University Obesity Solutions initiative, Arizona State University, Tempe, AZ 85287-2402 USA; 2 School of Human Evolution and Social Change, Arizona State University, Tempe, AZ 85287 USA

**Keywords:** Body image, Depression, Weight gain, Motherhood, Pregnancy, Postpartum, Obesity, MoBa, The Norwegian Mother and Child Cohort Study

## Abstract

**Background:**

Multiple studies show that obesity and depression tend to cluster in women. An “appearance concern” pathway has been proposed as one basic explanation of why higher weights might lead to depression. The transition to motherhood is a life phase in which women’s body image, weight, and depressive risk are in flux, with average weight increasing overall during this period. Examination of how these factors interact from pre- to post-pregnancy provides a means to test how body image plays a key role, as proposed, in causally shaping women’s depressive risk.

**Methods:**

Tracking 39,915 pregnant women in the Norwegian Mother and Child (MoBA) Cohort Study forward 36 months after their deliveries, we test the moderating and mediating effects of body image concerns on the emergence of new mothers’ depressive symptoms by using a binary logistic regression model with a discrete-time event history approach and mediation analysis with bootstrapping.

**Results:**

For women with high pre-pregnancy body mass index (BMI), weight gain heightens their depressive symptoms over time. Body image concerns mediate the association between weight gain and the development of depressive symptoms regardless of weight status. However, the mediation effect is more evident for women with higher pre-pregnancy BMI. Conversely, better body image is highly protective against the transition to mild or more severe depressive symptoms among new mothers, but only for women who were not classified as obese prior to their pregnancies.

**Conclusions:**

These findings support a role for body image concerns in the etiology of depressive symptoms during the transition to motherhood. The findings suggest body image interventions before or during pregnancy could help reduce risks of depression in the early postpartum period and well beyond.

## Background

The main goal of this study is to examine the proposed causal relationship between high body mass index (BMI) and the development of depressive symptoms after childbirth among Norwegian mothers, and to establish if negative body image accounts for this relationship. Multiple studies have demonstrated a general u-shaped relationship between BMI and depressive symptoms in women, with greatest risk observed in the heaviest women [1–3]. For example, a recent meta-analysis of community-based studies published between 1983 and 2007 across multiple countries concluded that women with obesity are approximately 32 % more likely to have depressive symptoms than their lower BMI female peers. Men’s depression, by contrast, appears little affected by high BMI [4].

Negative body image is associated with greater psychological distress regardless of gender [5–8]. For sociocultural reasons, women seem consistently more susceptible to having body image concerns than men [9]. Indeed, poor body image is one of a handful of variables that consistently predicts both obesity and depression outcomes [10–16]. Based on this finding, an “appearance concern” pathway is proposed to explain why obesity predicts depression in general, and why *weight gain* should then also worsen depression over time [13]. At present, the evidence supporting this proposed pathway is equivocal due to a lack of solid longitudinal analyses. Specifically, a limited number of studies show a significant association between body image concerns and self-esteem, though the direction of cause and effect is unclear [7, 17]. For example, Friedman et al. [12] tested two types of body image indices, measuring overweight men and women’s appearance and body satisfaction as mediators, and found significant partial mediation effects of both body image indices on depression and self-esteem. Gavin et al. [18] tested the mediation effect of a body image index in women aged between 40 and 65, measuring respondent satisfaction with her body shape by educational attainment, and concluded that body image exerts a significant mediating effect regardless of education level.

The transition from pre-pregnancy through the first 36 months is one in which body weights and depressive symptoms are in considerable flux, but both tend to increase overall [2, 19–21]. There is also some evidence that pre-pregnancy BMI matters to the emergence of depression during this time (hereafter glossed as the “extended postpartum”). A recent meta-analysis determined that women classified as obese (BMI ≥30) are 30 % more likely than women classified as normal weight (BMI between 18.5 and 25) to have depressive symptoms in the year following a birth [22]. One Danish longitudinal study (*N* = 70,355 women) showed that women whose BMI increased more between 6 months and 6 years postpartum relative to their pre-pregnancy values had more depressive or anxiety disorders (as defined by clinical diagnosis) compared to women who stayed nearer their pre-pregnancy BMI (difference in BMI between -1 and 1) [23]. More generally, body image concerns in and of themselves are associated with greater risk of depression among pregnant women and new mothers [24]; conversely, positive body image seems to be associated with higher self-esteem and myriad health benefits including better mental health [25]. In sum, previous studies suggest the likely importance of body image as a mediator explaining the association between weight status, weight gain, and depression among women, but solid evidence is yet to be provided.

Herein we present the results of a study that prospectively tracks Norwegian women through pregnancy and their first three years of motherhood, identifying how body image variables predict shifts in depressive status through time. A very large sample size allows us to clarify both women’s starting weight status and the influence of weight gained on the emergence of depressive symptoms. We hypothesize that women who have greater body image concerns prior to pregnancy and experience greater weight gain into motherhood will exhibit more depressive effects. Beyond better explicating how weight gain and body image are tied together in biosocial pathways to depression as part of a call to enhance understanding of the obesity-depression relationship in general [15], a more comprehensive model of how these factors interact is also needed to design effective depression intervention programs in the early postpartum period and on into motherhood [26, 27].

## Methods

### Study population

Our study uses data from the Norwegian Mother and Child Cohort Study (MoBa), a longitudinal study following more than 100,000 Norwegian women who were pregnant between 1999 and 2009 [28]. The first survey was conducted at the 17th week of pregnancy, then repeated at the 22nd and 30th weeks of pregnancy. In the first survey, respondents were asked about their current and pre-pregnancy weight and health status. After the birth of a child, respondents completed additional surveys when the child reached 6, 18, 36, and 60 months of age. For this study, we used data collected at the 17th week of pregnancy (time 0) and at 18 (time 1) and 36 (time 2) months after the birth of a child. As a result, we observe changes in weight and depressive symptoms during two time periods: between the 17th week of pregnancy (time 0) and 18 months postpartum (time 1) and between 18 (time 1) and 36 months postpartum (time 2). Since we are interested in how weight gain and body image concerns are associated with the development of depressive symptoms, we focused on the transition from few or no depressive symptoms to mild, moderate, or severe depressive symptoms. Accordingly, we focused our analysis on the subsample of respondents who did not report depression at the outset of pregnancy.

The initial available sample was 102,980 women. Because our focus is on the transition from baseline (time 0) to time points 1 and 2, we removed women who did not complete the questionnaires at times 0 and 1. Additionally, women who were pregnant at time 1 or 2 were excluded to avoid confounding of body weight/BMI data. Respondents with missing values in any variable or reporting implausible heights and weights (e.g., height <100 cm or >200 cm) were excluded, as were those with BMI below 18.5 (underweight). Finally, respondents who reported they had been diagnosed with or treated for depression pre-pregnancy at time 0 were removed. These criteria yielded a final sample of 39,915 (39 %) women. We then transformed this wide-format dataset to a long-format dataset in preparation for a discrete-time event history analysis, yielding 61,640 person-period cases. Secondary analysis of these data was approved by the Institutional Review Board of Arizona State University.

### Measures

#### Depressive symptoms

The severity of depressive symptoms was measured using a short version of the Hopkins Symptom Checklist (SCL-8) at time 1 and time 2 [29]. The SCL-8 asks, “have you ever been bothered by any of the following feelings during the past 2 weeks?” with eight items presented (e.g., “feeling fearful,” “feeling nervous or shaky inside,” “feeling hopeless about the future,” “feeling blue”) and a 4-point Likert scale from “not bothered” to “very bothered.” The depression score was calculated additively at each time point, so at times 1 and 2 scores range from 0 to 24. The standardized Cronbach Alpha coefficients show reliable internal consistency (.83 and .86 at times 1 and 2, respectively). Since depression points are right-skewed with many cases on the zero point, we defined four groups of depressive symptoms—few or no symptoms (0–5), mild (6–8), moderate (9–10), and severe (11–24)—following Chwastiak et al. [30]. Since our study focuses on the development of depressive symptoms after childbirth, the dependent variable is the transition from having no depressive symptoms at time 0 to having mild, moderate, or severe depressive symptoms at time 1 or 2.

#### Body image concerns

The questionnaires at 18 (time 1) and 36 (time 2) months postpartum used three sub-questions to measure body image concerns. The questions were, “have you during the last 6 months:”

1. “felt yourself that you were too fat?”
2. “been really afraid of putting on weight or becoming too fat?”
3. “felt that it was extremely important for your self-image to maintain a particular weight?”

For each question, a response of “yes” or “perhaps” was scored as +1. Points were summed to create a 0–4 point body image concern variable, where 4 was the highest level of concern. The standardized Cronbach Alpha coefficients show reliable internal consistency (.66 and .66 at times 1 and 2, respectively).

#### Weight change

The proportion of change in body mass index (BMI) was used to measure the weight change of a respondent over time. Weights and heights were self-reported and used to calculate BMI at times 0, 1, and 2. Mothers were then classified as normal/healthy (18.5 ≤ BMI < 25; hereafter referred to as “normal weight”), overweight (25 ≤ BMI < 30), or obese (30 ≤ BMI). BMI change refers to differences in BMI from the first time period (between the 17th week of pregnancy and 18 months after childbirth) to the second time period (between 18 and 36 months after childbirth). The proportion of change in BMI was then multiplied by 10, so that a 10 % change in BMI corresponds to a one-unit change in the variable. In addition, 25 cases with extreme weight changes between the two time periods are excluded. This variable is continuous and time-variant.

#### Covariates

We included a number of known covariates of postnatal depression in our models: lower socioeconomic status, worse health status, less exercise, more stressful life events and circumstances, less social support, and immigrant status [13, 15, 31–33]. To control for socioeconomic status we used level of education, parental income, and immigration status as covariates. Level of education is a dichotomous variable measuring whether or not a respondent finished or was enrolled in on-going higher education at the 17th week of pregnancy. Parental income is a categorical variable having the sum of both the mother and father’s incomes. It is grouped into three categories: low (combined income ≤450,000 NOK), medium, and high income (combined income ≥800,000 NOK). If a respondent or her child’s father spoke a language other than Norwegian, the respondent was coded as born outside Norway (i.e., immigrant). Immigration status is important to consider since immigrant women tend to have a higher chance of developing depressive symptoms in the extended postpartum compared to non-immigrant women [32]. Health status is a continuous variable summing the number of self-reported illnesses or health problems (excluding depression) a respondent reported prior to pregnancy. Respondents’ ages range from 18 to 47. Three age groups were created based on the age of a respondent at time 0: 18–29, 30–39, and 40–47. Two variables in the MoBa survey measure social support. The first variable used five questions related to various aspects of the respondent’s relationship with the father of her child at times 1 and 2. Each question has a maximum of five points, so the range of the final continuous, time-variant variable is between 0 and 25. The second time-variant variable measures how frequently a respondent communicated with close friends or family members, which we defined in three groups: talk rarely, talk occasionally, and talk frequently. In cases where a respondent checked two options, the response with a lower point or frequency was considered the correct answer to be conservative. Stress is a dichotomous variable indicating whether or not a respondent reported stressful life events, such as illness, hospitalization, or financial problems at each time point. We also created a variable measuring the intensity of physical activity in the respondent’s leisure time or at work to control for her level of physical activity before pregnancy. Since the physical activity question in time 2 was measured slightly differently from time 0 to time 1, the variable at time 2 is weighted so that it has the same possible range between 0 and 5.

### Statistical analysis

#### Step 1: Binary logistic regression

To test the associations among weight gain, body image concerns, and the development of depressive symptoms over time, we used a binary logistic regression model using a discrete-time event history approach (PROC LOGISTIC in SAS 9.4). This is the most appropriate technique given that the outcome variable is a dichotomous transition and that not all respondents experienced that transition. The duration of the event is one or two since our data has three time points (time 0, 1, and 2). By design, respondents become at risk of having depressive symptoms after pregnancy since all the respondents with depression before pregnancy are excluded from the sample, and they are removed from the risk once they develop mild or more severe depressive symptoms. And respondents who did not experience any depressive symptoms at all three time points are censored. For example, if a respondent who did not have depression before pregnancy reported mild depressive symptoms at time 1, she is censored at time 1 and the duration is one. If she reported no depressive symptoms at time 1, but did report symptoms at time 2, she is censored at time 2 and the duration is two. Therefore, the time unit of the analysis is the respondent-time. As shown in the example, each respondent can contribute as many as two cases to the sample. An additional consideration is the parameterization of time, which is measured with the time variable that indicates duration. The hazard has been parameterized with a dichotomous time variable having a value of one or two. Although event history analyses are typically used when there are many periods of risk (many years, many months), our analyses examine the transition across only two periods of risk. While this means there will be many ties in the data, the approach does not violate the assumptions of discrete-time event history, which is amenable to tied events.

In this binary logistic regression model, the interaction between body image concerns and the development of depressive symptoms is tested as well. The interaction results, if significant, tell us how the total effect of weight change on the development of depressive symptoms varies at each level of body image concerns [34, 35].

#### Step 2: Mediation analysis with bootstrapping

In the next step, we assessed the effects of weight change on depressive symptoms through body image concerns. The results tell us how body image concerns mediate the total effect of weight change on depressive symptoms when the effect exists [34, 35]. The most widely used method for mediation analysis is the causal four steps approach by Baron and Kenny [34]. Though intuitive and effective to test mediation, the approach has some limitations including the imbalance of confidence limits due to the non-normality of the distribution of the indirect effect [36]. MacKinnon, Lockwood, and Williams [37] suggest two methods to overcome this limitation, and their simulation study confirms that confidence limits are the most accurate when a bias-corrected bootstrap resampling method is used. Further, the method performs fairly well when the outcome is dichotomous, like our dependent variable, the transition to mild or more severe depressive symptoms. However, our mediator, body image concerns, and the main independent variable, weight change, are both time-varying, which is likely to cause some biases for confidence limits. To circumvent this issue, we calculated the confidence limits of the indirect effect of body image concerns for each time period separately. We used the user-made *binary_mediation* command in Stata with the bootstrap command with 5,000 sampling replications following Jackson, Beeken, and Wardle [38]. This analysis enables us to estimate direct, indirect, and total effects; the bootstrap command is used to obtain the bias-corrected 95 % confidence intervals of the coefficients for those three effects in each time period.

## Results

Tables 1 and 2 summarize the descriptive statistics for the variables used in the analysis for two time periods (times 1 and 2) by initial weight status (time 0). The descriptive statistics of the total 39,915 respondents who completed the survey at times 0 and 1, and who did not have depression at time 0, are summarized in (Table 1). In (Table 2), the descriptive statistics of the total 21,715 respondents (=14,961 + 4,839 + 1,925) who were not censored (i.e., who did not report depressive symptoms) between times 0 and 1 are summarized.

**Table 1:** Summary statistics for the time period between the 17^th^ Week of pregnancy (time 0) and the 18^th^ Month Postpartum (time 1)

	Normal weight (18.5 < BMI ≤ 25)				Overweight (25 < BMI ≤ 30)				Obese (30 < BMI)			
	Mean	S.D.	Min.	Max.	Mean	S.D.	Min.	Max.	Mean	S.D.	Min.	Max.
*Transition to mild or more severe depressive symptoms (1/0)*												
between time 0 and 1	0.09	0.29	0	1	0.10	0.29	0	1	0.11	0.32	0	1
*Time-variant variables at Time 1*												
BMI change	0.21	0.64	-3.57	8.33	0.11	0.73	-5.73	4.29	-0.10	0.82	-4.09	3.78
Body image concern	1.48	1.15	0	3	2.07	0.95	0	3	2.19	0.86	0	3
Partner	20.56	4.11	0	25	20.44	4.21	0	25	20.22	4.43	0	25
Talk rarely	0.01	0.10	0	1	0.01	0.10	0	1	0.01	0.12	0	1
Talk occasionally	0.22	0.41	0	1	0.20	0.40	0	1	0.17	0.38	0	1
Talk often	0.77	0.42	0	1	0.79	0.41	0	1	0.81	0.39	0	1
Stress	0.98	0.15	0	1	0.97	0.16	0	1	0.96	0.19	0	1
Exercise	2.63	1.37	0	5	2.74	1.38	0	5	2.82	1.39	0	5
*Time-invariant variables at Time 0*												
Health	0.96	1.16	0	10	1.07	1.24	0	8	1.22	1.34	0	10
Higher education	0.72	0.45	0	1	0.64	0.48	0	1	0.53	0.50	0	1
Low income	0.09	0.28	0	1	0.09	0.28	0	1	0.11	0.32	0	1
Mid income	0.75	0.43	0	1	0.80	0.40	0	1	0.81	0.39	0	1
High income	0.16	0.37	0	1	0.12	0.32	0	1	0.07	0.26	0	1
Foreign-born	0.10	0.31	0	1	0.08	0.27	0	1	0.07	0.26	0	1
In the 20s	0.40	0.49	0	1	0.39	0.49	0	1	0.41	0.49	0	1
In the 30s	0.58	0.49	0	1	0.58	0.49	0	1	0.56	0.50	0	1
In the 40s	0.02	0.14	0	1	0.02	0.15	0	1	0.03	0.16	0	1
Twins or triplets	0.02	0.13	0	1	0.02	0.13	0	1	0.02	0.14	0	1
Age	30.60	4.23	18	47	30.75	4.39	18	47	30.59	4.50	18	46
N	27,339				8845				3731

**Table 2:** Summary statistics for the time period between the 18^th^ month postpartum (time 1) and the 36^th^ month postpartum (time 2)

	Normal weight (18.5 < BMI ≤ 25)				Overweight (25 < BMI ≤ 30)				Obese (30 < BMI)			
	Mean	S.D.	Min.	Max.	Mean	S.D.	Min.	Max.	Mean	S.D.	Min.	Max.
*Transition to mild or more severe depressive symptoms (1/0)*												
between time 1 and 2	0.06	0.25	0	1	0.06	0.24	0	1	0.07	0.26	0	1
*Time-variant variables at Time 2*												
BMI change	0.11	0.57	-3.98	6.94	0.07	0.65	-3.30	3.67	0.08	0.80	-4.35	3.64
Body image concern	1.25	1.11	0	3	1.78	0.97	0	3	1.91	0.89	0	3
Partner	20.35	4.22	0	25	20.28	4.27	0	25	20.08	4.31	1	25
Talk rarely	0.01	0.09	0	1	0.01	0.10	0	1	0.01	0.10	0	1
Talk occasionally	0.23	0.42	0	1	0.22	0.42	0	1	0.20	0.40	0	1
Talk often	0.77	0.42	0	1	0.77	0.42	0	1	0.79	0.41	0	1
Stress	0.43	0.50	0	1	0.50	0.50	0	1	0.54	0.50	0	1
Exercise	1.44	0.97	0	5	1.49	0.97	0	5	1.45	1.01	0	5
*Time-invariant variables at Time 0*												
Health	0.93	1.12	0	10	1.03	1.19	0	8	1.17	1.28	0	8
Higher education	0.74	0.44	0	1	0.67	0.47	0	1	0.57	0.50	0	1
Low income	0.07	0.26	0	1	0.07	0.26	0	1	0.10	0.30	0	1
Mid income	0.75	0.43	0	1	0.80	0.40	0	1	0.81	0.39	0	1
High income	0.17	0.38	0	1	0.12	0.33	0	1	0.09	0.28	0	1
Foreign-born	0.09	0.29	0	1	0.07	0.26	0	1	0.07	0.25	0	1
In the 20s	0.37	0.48	0	1	0.36	0.48	0	1	0.37	0.48	0	1
In the 30s	0.61	0.49	0	1	0.61	0.49	0	1	0.61	0.49	0	1
In the 40s	0.02	0.14	0	1	0.03	0.16	0	1	0.03	0.16	0	1
Twins or triplets	0.02	0.13	0	1	0.02	0.14	0	1	0.02	0.14	0	1
Age	30.95	4.14	18	47	31.09	4.32	18	47	30.96	4.33	18	46
N	14,961				4839				1925			

In detail, 9.1 % of respondents (2492 of 27,339 respondents) with BMI between 18.5 and 25 (normal weight), 9.6 % of respondents (850 of 8845 respondents) with BMI between 25 and 30 (overweight), and 11.2 % of respondents (417 of 3,731 respondents) with BMI higher than 30 (obese) developed mild or more severe depressive symptoms between times 0 and 1. Among the respondents who did not develop mild or more severe depressive symptoms between times 0 and 1,6.4 % of respondents classified as normal weight (962 out of 14,961 respondents), 6.0 % of respondents classified as overweight (295 out of 4839 respondents), and 7.1 % of respondents classified as obese (137 out of 1925 respondents) developed mild or more severe depressive symptoms between times 1 and 2. In both time periods, more overweight and obese women experienced depressive symptoms over time compared to women with normal weights.

In addition, overweight and obese women tended to have poorer body image than women with normal BMI between times 0 and 1. This pattern was repeated between times 1 and 2, though the level of body image concerns for those respondents who did not develop mild or more severe depressive symptoms between times 0 and 1 was slightly lower (better) than those respondents who were censored between time 0 and time 1. Further, overweight and obese women communicated more actively with their close friends or family members (not including spouses or life partners) than did women with normal BMI. BMI did not change substantially over time for any of the three groups, but overweight and obese women tended to exercise slightly more often than women with normal BMI. Women classified as overweight and obese were less educated, earned less, and had more illnesses or health problems than women classified with normal BMI.

### Step 1: Binary logistic regression results

In the first step, we ran a series of binary logistic regressions using a discrete-time event history model approach by initial weight status to estimate the effects of BMI change and body image concerns on the transition from having no depressive symptoms to experiencing some degree of depressive symptoms. The results for all three groups are summarized in (Table 3). Model 1 includes only BMI change, model 2 includes both BMI change and body image concerns, and model 3 includes the interaction term between the two. The results are presented as odds ratios, so a coefficient greater than one represents a positive effect that accelerates the rate of the transition, while a coefficient less than one represents a negative effect that delays the transition.

**Table 3:** Discrete-time event history model results

	Model 1			Model 2			Model 3		
	Odds Ratio	95 % C.I.	*p-value*	Odds ratio	95 % C.I.	*p-value*	Odds ratio	95 % C.I.	*p-value*
*Normal weight*									
BMI change	1.037	(0.980; 1.098)	0.210	0.952	(0.898; 1.010)	0.104	0.755	(0.671; 0.849)	<.0001
Body image concern				1.395	(1.348; 1.443)	<.0001	1.372	(1.326; 1.421)	<.0001
BMI change * Body image concern							1.128	(1.070; 1.189)	<.0001
-2 Log Likelihood	21188.81			20803.72			20783.46		
Person-time	42,300								
*Overweight*									
BMI change	1.043	(0.956; 1.138)	0.344	0.997	(0.913; 1.089)	0.947	0.681	(0.519; 0.894)	0.006
Body image concern				1.650	(1.525; 1.784)	<.0001	1.644	(1.520; 1.778)	<.0001
BMI change * Body image concern							1.171	(1.053; 1.303)	0.004
-2 Log Likelihood	6923.74			6749.73			6741.34		
Person-time	13,684								
*Obese*									
BMI change	1.178	(1.053; 1.319)	0.004	1.163	(1.040; 1.302)	0.008	1.047	(0.903; 1.208)	0.808
Body image concern				1.563	(1.380; 1.770)	<.0001	1.561	(1.379; 1.768)	<.0001
BMI change * Body image concern							1.045	(0.903; 1.208)	0.557
-2 Log Likelihood	3152.89			3099.47			3099.13		
Person-time	5656								

Note: C.I. stands for confidence intervals (Wald). The variables to measure social support, stress, exercise, health, education, income, age, and singleton or not are controlled for in each model but omitted

For women with normal weights, a one-unit (10 %) increase in BMI over time does not change the likelihood of the transition from no depressive symptoms to mild or more severe depressive symptoms. This result is identical for overweight women. For women classified as obese, however, a 10 % increase in BMI significantly increases the likelihood of the transition to experiencing depressive symptoms by 18 % in model 1. It should be noted that a one-unit increase in BMI, which is a 10 % increase in BMI compared to the previous time point, requires greater overall weight gain for women starting with higher body weights. For example, a woman who weighs 95 kg (about 209 lbs) and measures 1.7 m (about 5 ft 7 in) in height would have to gain 9.5 kg (about 21 lbs) for her BMI to increase by 10 %. By comparison, a woman of the same height with a starting weight of 60 kg only needs to gain 6 kg.

The results for body image concerns in model 2 for all three weight status groups, unlike those for BMI change, show significant associations with the likelihood of having depressive symptoms. For example, for obese women, a one-unit increase in body image concerns significantly increases the likelihood of having depressive symptoms by 56 %. The size of the effect varies for women who are normal weight and overweight, but the directions are the same.

The interaction between BMI change and body image concerns is significant for women who are classified as normal weight and overweight. The results indicate that normal weight and overweight women are more likely to experience depressive symptoms when they have more body concerns. The results for women categorized as obese, however, do not show any significant interaction effects.

Focusing on model 2 across tables, the results of the socioeconomic and demographic factors show similar patterns to previous studies across all three weight status groups, with some exceptions [27, 31] (results not provided). As expected, more social support, higher income, more education, fewer health problems, and fewer adverse life events were associated with fewer depressive symptoms.

### Step 2: Mediation analysis results

To test if body image concerns mediate the effect of BMI change on developing mild or more severe depressive symptoms, mediation analysis was conducted for two time periods separately in the final step of the analysis. Previous studies showed that the periods during pregnancy and immediately following birth are crucial for the development of depression [33, 39], but it is also likely that BMI change has a long-term effect on depression and anxiety as well across the extended postpartum [23]. The results of the mediation analysis are summarized in (Table 4).

**Table 4:** Mediation analysis results of the association between weight gain and experiencing depressive symptoms with body image concerns as a mediator

	Between time 0 and 1			Between time 1 and 2		
	Coefficient	Bootstrap S.E.	Bias-corrected 95 % C.I.	Coefficient	Bootstrap S.E.	Bias-corrected 95 % C.I.
*Normal weight*						
Indirect effect (via mediator)	**0.0309**	0.0023	(0.0265; 0.0357)	**0.0161**	0.0022	(0.0121; -0.0207)
Direct effect	0.0019	0.0136	(-0.0236; 0.0289)	**-0.0748**	0.0197	(-0.1126; -0.0350)
Total effect	**0.0328**	0.0136	(0.0074; 0.0599)	**-0.0587**	0.0197	(-0.0967; -0.0193)
# replications	5000			4999		
*Overweight*						
Indirect effect (via mediator)	**0.0242**	0.0036	(0.0176; 0.0317)	**0.0132**	0.0040	(0.0063; -0.0220)
Direct effect	-0.0313	0.0228	(-0.0755; 0.0141)	**0.0870**	0.0315	(0.0227; -0.1451)
Total effect	-0.0072	0.0232	(-0.0524; 0.0389)	**0.1001**	0.0315	(0.0363; -0.1595)
# replications	4999			4960		
*Obese*						
Indirect effect (via mediator)	**0.0080**	0.0038	(0.0017; 0.0169)	0.0088	0.0062	(-0.0012; -0.0239)
Direct effect	0.0593	0.0316	(-0.0044; 0.1190)	0.0777	0.0526	(-0.0325; -0.1739)
Total effect	**0.0673**	0.0318	(0.0049; 0.1277)	0.0865	0.0529	(-0.0251; -0.1838)
# replications	4999			3017		

Note: **Bold** if the result is significant at least at the .05 level of significance; S.E. stand for standard errors; C.I. stands for confidence intervals. The variables to measure social support, stress, exercise, health, education, income, age, and singleton or not are controlled for in each model but omitted

Between time 0 (17^th^ week of pregnancy) and time 1 (18 months after childbirth), the results show a significant and positive indirect effect of body image concerns on the association between BMI change and depressive symptoms. The effect is obvious regardless of the mother’s body size (BMI). However, we confirmed that the interactions between weight gain and body image concerns are significant for normal weight and overweight women (Table 3), but not for women classified as obese. This finding suggests that the effects of weight gain on experiencing depressive symptoms vary at different levels of body image concerns for women with smaller body sizes. Focusing on the women with obesity, a group that did not show a significant moderating effect of body image concerns, the results indicate a significant indirect effect. In detail, a 10 % increase in BMI between times 0 and 1 is associated with a roughly. Eight percent increase in the likelihood of having mild or more severe depressive symptoms mediated by body image concerns (exp (.0080) = 1.008). Body image concerns account for about 12 % of the total effect (.0080/.0673 = .12). In other words, about 12 % of the total effect of BMI change on the development of depressive symptoms up to 18 months postpartum can be explained by women’s negative body image concerns.

In the second time period between the 18^th^ month and the 36^th^ month after childbirth (between times 1 and 2), however, the results are more mixed. For those women with obesity, the indirect effect is not significant in the second time period. Yet, indirect effects are still significant for normal weight and overweight women. However, the results for women classified as normal BMI show inconsistent mediation [36]. The total effect indicates that for mothers classified with normal BMI, BMI change is negatively associated with the development of mild or more severe depressive symptoms (negative). However, the indirect effect has a positive sign, meaning that among those respondents who did not develop mild or more severe depressive symptoms in the first time period, in the second time period (1) BMI gain was positively associated with higher levels of body image concerns, and (2) body image concerns were also positively associated with the likelihood of developing mild or more severe depressive symptoms (separate test results can be provided upon request).

## Discussion

This study contributes to our growing understanding of the role of body image in the complex relationship between obesity and depression, with specific focus on the changes in these three factors during the extended transition into motherhood. The very large cohort sample and longitudinal nature of the dataset are advantages of this study. Our results are novel in that they provide increased clarity on some key proposed relationships between women’s body image, weight change, weight status, and depression in the first three years after women give birth. Consistent with expectations and previous studies [40], we show a significant association between poor body image and the emergence of depressive symptoms over time.

The study thus supports two basic conclusions about the role of body image concerns in the emergence of depressive symptoms. First, it provides a clearer picture of how women with higher BMI have a greater risk of developing depressive symptoms across the extended postpartum period. For these women, substantial further gains in weight following pregnancy can tip them toward or into developing depressive symptoms. Our mediation analysis supports the conclusion that body image concerns also partially explain how weight change in women with pre-pregnancy obesity affects the development of depressive symptoms, especially in the first 18 months after childbirth.

Second, our study adds to our understanding about how new normal weight and overweight (but not obese) mothers develop depressive symptoms. The interaction results suggest that for women who had normal weight and overweight bodies pre-pregnancy—even those who experience significant weight change—having a positive body image is highly protective against a transition to more depressive symptoms in the years after birth. Conversely, normal weight and overweight women who had worse body image and gained weight are more likely subsequently to develop depressive symptoms. Body image concerns accordingly affect all women, regardless of weight status, even if women with the highest pre-pregnancy body weights are especially vulnerable to their effects.

Our observation that positive body image is protective against the development of depressive symptoms for all groups except obese women suggests the role of body image is different for women classified pre-pregnancy as normal or overweight versus obese. One possible explanation is that a longer history of internalized stigma related to feeling judged because of weight, felt more acutely by women who begin the transition to motherhood already with high body weights [9], is more important to women’s susceptibility to later emergence of depressive symptoms than actual levels of weight gain during pregnancy or following a birth than weight *per se*. The findings provide additional support to the proposition that body image concerns are important to explaining why women’s depressive symptoms are better predicted by body weights than are men’s. In general, the results also support the appearance pathway model proposed by Makowitz et al. [13], which suggests a meaningful role for body image concerns on the development of depression more generally.

The Norwegian Mother and Child Cohort Study (MoBA) is a large, high-quality prospective dataset that provides the very large sample that is the major strength of this study, allowing modeling of complex relationships among interacting factors to identify effects not so readily observable in smaller-scale or cross-sectional studies. A particular strength of this study is that we consider the fact that body image concerns can change through time and across the motherhood transition in both positive and negative directions, just as can depressive symptoms and weight. Primary limitations of the study are related to our variable and model construction. First, the measurement of body image concerns is constructed based on dichotomous answers from few items. As a result, the reliability test results for the body image concerns index are within an acceptable range but not ideal. Second, the lagged effects of body image concerns on depressive symptoms could not be tested. Body image concerns were measured at times 1 and 2, and they predicted depressive symptoms at times 1 and 2, respectively. This means we can suggest, but cannot clearly demonstrate, a cause-and-effect relationship between body image and depression [24]. But we also note that the effect size for the association is relatively smaller when longitudinal data with lagged effects is analyzed compared to cross-sectional studies [26]. A further limitation is the use of self-reported weight and height data, rather than direct measurements.

## Conclusion

These findings support a substantial role for body image concerns in the etiology of women’s depressive symptoms in their reproductive years, at least associated with the transition to motherhood. More generally, it reinforces the notion that body image is a broad public health concern. It can act as a significant driver of women’s mental health and, in the contexts of early motherhood, perhaps be a little-observed but potentially significant factor shaping children’s health as well, given that maternal depression places both women *and their children* at considerably greater risk of a range of adverse social, economic, and health outcomes. These findings highlight the need for translational research that assesses the feasibility and efficacy of body image interventions for improving the health of both groups [1, 40]. While this study shows that body image concerns can most substantially influence the risk of subsequently developing depressive symptoms for women who begin their pregnancies with higher body weights, women at all body sizes also demonstrate significant effects. Thus, interventions to improve body image could benefit all women, regardless of their weight status.
